# Three-dimensional cancer cell migration directed by dual mechanochemical guidance

**DOI:** 10.1103/physrevresearch.4.l022007

**Published:** 2022-04-12

**Authors:** Pedram Esfahani, Herbert Levine, Mrinmoy Mukherjee, Bo Sun

**Affiliations:** 1 Department of Physics, Oregon State University, Corvallis, Oregon 97331, USA; 2 Center for Theoretical Biological Physics, Northeastern University, Boston, Massachusetts 02115, USA; 3 Departments of Physics and Bioengineering, Northeastern University, Boston, Massachusetts 02115, USA

## Abstract

Directed cell migration guided by external cues plays a central role in many physiological and pathophysiological processes. The microenvironment of cells often simultaneously contains various cues and the motility response of cells to multiplexed guidance is poorly understood. Here we combine experiments and mathematical models to study the three-dimensional migration of breast cancer cells in the presence of both contact guidance and a chemoattractant gradient. We find that the chemotaxis of cells is complicated by the presence of contact guidance as the microstructure of extracellular matrix (ECM) vary spatially. In the presence of dual guidance, the impact of ECM alignment is determined externally by the coherence of ECM fibers and internally by cell mechanosensing Rho/Rock pathways. When contact guidance is parallel to the chemical gradient, coherent ECM fibers significantly increase the efficiency of chemotaxis. When contact guidance is perpendicular to the chemical gradient, cells exploit the ECM disorder to locate paths for chemotaxis. Our results underscore the importance of fully characterizing the cancer cell microenvironment in order to better understand invasion and metastasis.

## I. INTRODUCTION

Directed cell migration [1] is of fundamental importance in wound healing [2], immune response [3], and cancer metastasis [4]. In these processes, cells are presented with various types of extracellular cues that bias the direction of their otherwise random motion. Chemotaxis is a major type of chemical guidance [5,6] that drives a cell to follow the gradient of chemoattractants in orchestrated functions of multicellular organisms [7,8].

Contact guidance, on the other hand, is a mechanical effect that directs cell morphogenesis and motility based on cues from the topography of two-dimensional (2D) substrates [9,10] or the organization of 3D extracellular matrix [11]. Unlike chemotaxis, contact guidance presents a nematic cue to the cells [12]. Migration modulated by contact guidance is observed in many cell types [13-16], where cells directed by contact guidance are also considered to follow a least-resistance path [17,18].

While the motility of cells under either chemotaxis or contact guidance has been extensively characterized, in realistic physiological conditions chemical cues and mechanical guidance are often present simultaneously. During tumor progression, chemotaxis driven by the gradient of various growth factors and nutrients facilitates the dissemination of cancer cells from primary to metastatic sites [19]. To finish this journey, cancer cells must navigate through the host tissue space filled by fibrous extracellular matrix (ECM) [20], with the alignment of ECM fibers as a major source of contact guidance. In fact, it has been shown that the direction of ECM fibers has a significant correlation with tumor prognosis [16,21].

The migrational response by which a cancer cell integrates multiplexed cues remains poorly understood [22]. The issue is particularly relevant for 3D cell migration, as many widely used chemotaxis assays do not account for the effects of ECM alignment. We expect the overlooked aspects of mechanochemical dual guidance will translate to *in vivo* processes including development and metastasis. Addressing the knowledge gap, here we report a combined experimental and theoretical analysis to elucidate 3D breast cancer cell motility under joint control of mechanical and chemical cues. We show that both ECM microstructure and cell mechanotransduction modulate cancer cell chemotaxis. Despite the complexity of the underlying molecular mechanisms, our results support a simple picture whereby chemotaxis and contact guidance provide additive motility biases to steer 3D cell migration. Taken together, our results underscore the importance of fully characterizing the cancer cell microenvironment in order to better understand invasion and metastasis.

## II. RESULTS

**FIG. 1.**
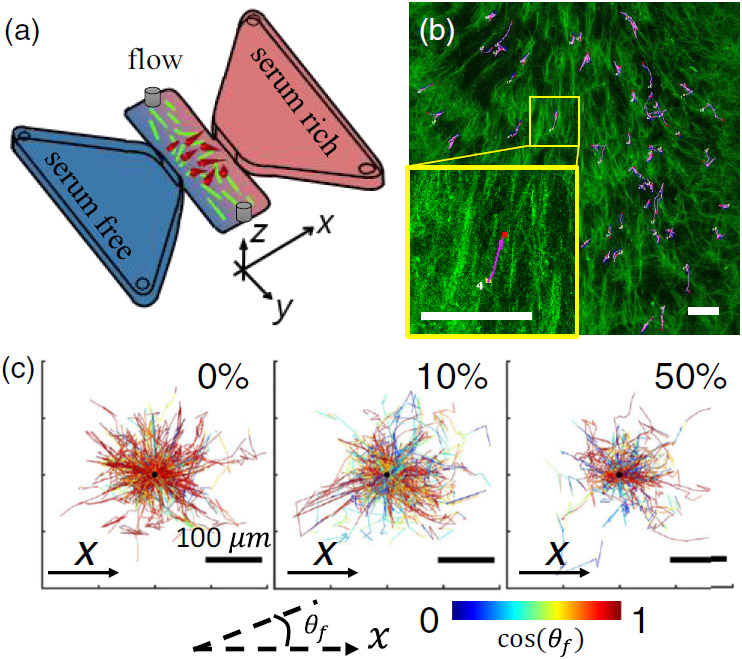
Experimental setup used to investigate 3D cancer cell migration in the presence of dual mechanochemical guidance. (a) Schematics of the dual guidance assay. (b) Example cell trajectory (purple) over collagen ECM (green). The yellow box shows a zoomed-in view of a cell following aligned collagen fibers. To compute the direction and coherence of the ECM fiber surrounding a cell, a 150 *μ*m × 150 *μ*m observing window is positioned around the cell of interest. (c) Displacement of cancer cells in the *x-y* plane under various chemogradient conditions. Here 0%, 10%, and 50% represent the volume concentration of serum in the chemoattractant reservoir, while the other reservoir is filled with serum-free growth medium. The trajectories are colored by the cues agreement cos $\theta_{f}$. Scale bars: 100 *μ*m.

In order to investigate 3D cancer cell motility in the presence of simultaneous mechanical and chemical guidance, we incorporate flow-induced ECM alignment in 3D chemotaxis assays. In particular, we inject 1 mg/mL neutralized, FITC-labeled type-I collagen solution into the 3D cell migration chamber [Fig. 1(a), along the $\hat{y}$ direction [23]]. The collagen solution contains low-density RFP-labeled MDA-MB-231 cells and gradually gelates to form fibrous collagen ECM. Using a syringe pump, we program the injection speed and duration and also alternate the flow direction. The resulting flow field directs the self-assembly of collagen ECM, thereby creating a network of collagen fibers whose geometry vary spatially. Once the ECM is set, we fill the liquid reservoirs with serum-free and serum-rich (10% or 50% volume concentration and 0% as control) growth media. In less than 6 h, a stable gradient is formed across the migration chamber, which measures 1 mm between the two reservoirs. We conduct live cell confocal imaging of both cells and collagen fibers 6 h after device setup. Each device is continuously imaged for 12 h at intervals of 15 min, where *z* stacks at steps of 10 *μ*m are collected [24]. Because both serum gradient and collagen fiber alignment are primarily in the *x-y* plane, we focus on the cell motility in the *x-y* projection. Specifically, the chemical gradient drives the cells toward $+\hat{x}$ direction, whereas the local mechanical guidance may point along any direction. We track cell centroids and estimate cell velocity from the displacement over 30 min [25,26].

The ECM is intrinsically a disordered biopolymer network whose microstructure fluctuates spatially [27]. Therefore, instead of bulk characterizations, we must quantify the local contact guidance cues. To this end, we employ image-based methods to measure the principal direction and coherence (*c*) of collagen fibers in subwindows surrounding individual cells, as reported previously [12,28] [Fig. 1(b)]. Each subwindow is a cell-centered 150 *μ*m × 150 μm square, cropped from the same *z* slice in which the cell is mostly in focus. Coherence *c* varies between 0 and 1, with 0 for completely random aligned fibers and 1 for perfectly parallel fibers. In our experiments, local coherence typically varies between 0.1 and 0.4. The approach of using cell-centered subimage characterization allows us to compute the instantaneous contact guidance experienced by individual cells.

As a cancer cell navigates the ECM under dual guidance, cos $\theta_{f}$, where $\theta_{f}$ is the angle between the principal direction of collagen fibers and $\hat{x}$ axis, represents the cues agreement. Since contact guidance is nematic, we only need to consider the acute angle with $|\theta_{f}|\leq \frac{\pi }{2}$. When cos $\theta_{f}=1$, contact guidance is parallel with the chemical gradient and we expect both cues to work in concert to facilitate chemotactic motion. When cos $\theta_{f}=0$, on the other hand, local contact guidance is perpendicular to the chemical gradient. In this case, we expect mechanical and chemical guidance to be in strongest competition for steering the direction of cell migration. In a typical cell trajectory [Fig. 1(b)] the direction of contact guidance varies along the cell’s migration path. This effect is further demonstrated in Fig. 1(c), which shows trajectories (offset by their starting position) of a random subset of cells in different magnitudes of chemical gradient. The trajectories are color coded by cues agreement cos $\theta_{f}$. It is evident that, while overall a positive chemical gradient biases the cell motility towards serum-rich region, heterogeneous mechanical guidance complicates the task of chemotaxis.

**FIG. 2.**
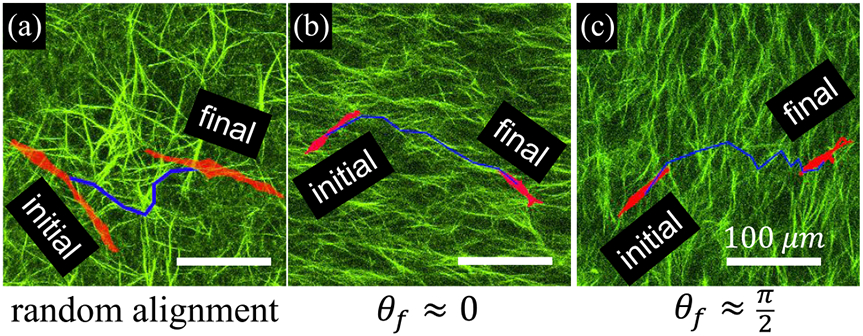
Cancer cell motility integrates dual mechanochemical guidance by exploiting the disordered fibrous structure of the ECM to move up chemogradients. Examples shown here are (a) a cancer cell moves in a region of randomly aligned ECM, (b) a cancer cell moves along ECM aligned parallel to the chemogradient, and (c) a cancer cell takes advantage of ECM disorder to traverse collagen fibers aligned perpendicular to the chemogradient. Scale bars: 100 *μ*m. Red: morphology of cells at the initial and final frames. Green: collagen fibers. Blue lines: cell trajectories.

By more closely examining the cell migration in dual guidance, we notice characteristics of the path-finding dynamics. As illustrated in Fig. 2, a cancer cell typically searches for collagen fibers that lie in the direction dictated by chemotaxis. When local coherence is low [Fig. 2(a)], such fibers are possible to find because collagen fibers are randomly aligned. Conversely, when local fibers primarily align in the direction of chemical gradient [Fig. 2(b)], cells can readily follow the contact guidance to move towards higher serum concentration. Interestingly, even when collagen fibers are mostly aligned perpendicular to the gradient [Fig. 2(c)], cancer cells are capable of locating fibers in the direction of chemical guidance. Such fibers are rare in this case; therefore, cells spend much time waiting, or moving perpendicular to the chemical cue until a path suitable for chemotactic progress is discovered [29].

To better quantify these observations, we propose a simple mathematical model to understand how cells integrate simultaneous mechanochemical guidance. In particular, we consider the direction $\theta_{v}$ of cell velocity to follow the Langevin equation, or its equivalent drift-diffusion equation that governs $P(\theta_{v},t)$, the probability distribution of $\theta_{v}$:

(1) $$\frac{d\theta_{v}}{dt}=-\alpha \;\sin\;\theta_{v}-\beta \;\sin\;2(\theta_{v}-\theta_{f})+\eta ,$$

(2) $$\frac{\partial \;P(\theta_{v},t)}{\partial t}=\frac{\partial }{\partial \theta_{v}}([\alpha \;\sin\;\theta_{v}+\beta \;\sin\;2(\theta_{v}-\theta_{f})]P(\theta_{v},t))\;\;+D\frac{\partial^{2}}{\partial \theta_{v}^{2}}P(\theta_{v},t).$$

Here *α* > 0 represents the $\hat{x}$-directional driving by the chemical guidance, *β* > 0 represents the effects of contact guidance, and *η* is a Gaussian random force representing noise whose amplitude is controlled by an effective diffusion coefficient *D* such that $\langle \eta (t)\eta (t^{\prime })\rangle =2D\delta (t-t^{\prime })$. Equation (1) assumes cancer cells additively integrate the chemical and mechanical cues, under the impact of intrinsic and extrinsic noises. Note that, while chemical guidance is unidirectional (invariant under $\theta_{v}\to \theta_{v}+2\pi$), contact guidance is nematic (invariant under $\theta_{v}\to \theta_{v}+\pi$ or $\theta_{f}\to \theta_{f}+\pi$).

When coherence *c* is low, we expect $\frac{\beta }{\alpha }$ to be small. In this case, cell chemotaxis will navigate randomly aligned fibers regardless of the principal direction of the local ECM. When *c* is higher, the direction of collagen fibers $\theta_{f}$ will have a strong impact of cell motility. Chemotaxis is enhanced when cues agreement is high, as cell path finding becomes easier. Conversely, chemotaxis is curtailed when cues agreement is low, as there are fewer fibers along the chemogradient. Therefore, we expect *β* to be positively related with coherence *c*.

We can solve Eq. (1) to obtain the stationary distribution of $\theta_{v}$

(3) $$P_{ss}(\theta_{v})=N\;\text{exp}\left[\frac{\alpha }{D}\;\cos\;\theta_{v}+\frac{\beta }{D}\;\cos\;2(\theta_{v}-\theta_{f})\right],$$

where *N* is a normalization factor.

**FIG. 3.**
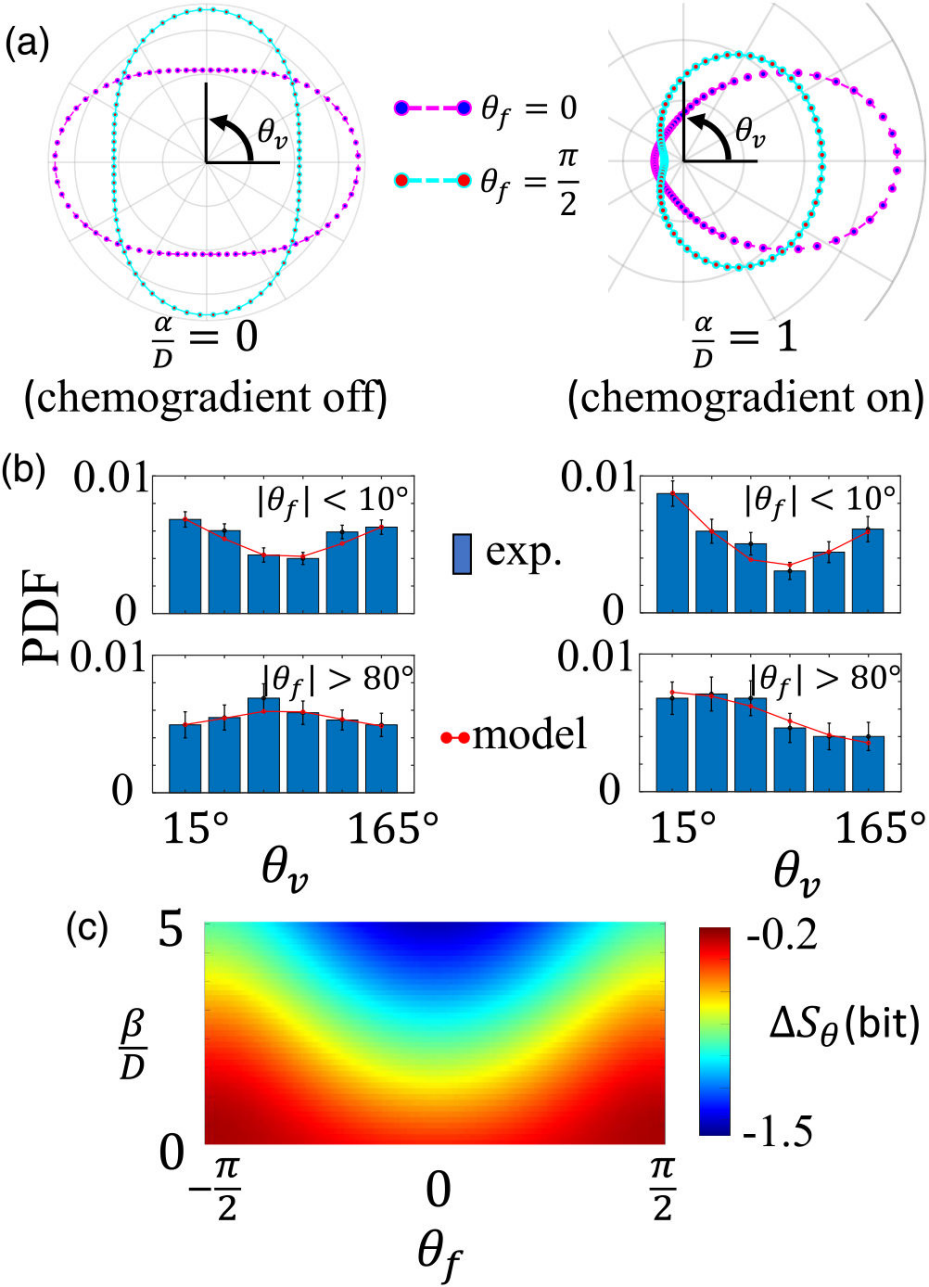
Theoretical model predicts cancer cell motility in response to dual mechanochemical guidance. (a) Probability distribution of cell velocity direction ($\theta_{v}$) when cues agreement is maximal (magenta) and when cues agreement is minimal (cyan). Left: only the mechanical cue is present. Right: both chemical and mechanical cues are present. In these examples we choose $\frac{\beta }{D}=0.5$. (b) Typical experimental results corresponding to scenarios shown in (a). Abbreviations: PDF, probability distribution function; exp., experiments. For each histogram, *N* > 1000 data points are included. Error bars are obtained from standard deviation of 100 bootstrap iterations. (c) Uncertainty of cell migration direction quantified by the differential entropy of $\theta_{v}$. The heat map shows the change of differential entropy $\Delta S_{\theta }$ in comparison with the case without external guidance (*α* = *β* = 0). The results in (c) are calculated based on the model by setting $\frac{\alpha }{D}=1$.

To illustrate the model results, we choose generic parameters and consider four distinct scenarios [Fig. 3(a)]. In the absence of a chemogradient ($\frac{\alpha }{D}=0$), the direction of cell migration is biased solely by the ECM direction. The probability distribution function (PDF hereafter) of $\theta_{v}$ reflects the value of $\theta_{f}$ and is symmetric with respect to the line $\theta_{v}=\theta_{f}$. Also in this case $P_{ss}(\theta_{v})=P_{ss}(\theta_{v}+\pi )$, because contact guidance alone is a nematic cue to cell motility.

Once we turn on chemogradient ($\frac{\alpha }{D}>0$), $P_{ss}(\theta_{v})$ shifts toward $+\hat{x}$ direction. It is informative to consider two special cases with maximal cues agreement $\theta_{f}=0$ and minimal cues agreement $\theta_{f}=\frac{\pi }{2}$. PDF of $\theta_{v}$ is symmetric with respect to the *x* axis, which is again due to the nematic nature of contact guidance. It is evident that chemotaxis index CI, defined by ⟨$\cos\;\theta_{v}$⟩, is positive. In general, we note that Eq. (3) is invariant under reflection symmetry ($\theta_{f}\to -\theta_{f},\theta_{v}\to -\theta_{v}$) and nematic symmetry ($\theta_{f}\to \theta_{f}+\pi$). These symmetries are model independent.

The four scenarios (with and without chemogradient at maximum or minimum cues agreement), along with the model predicted PDF of cell migration direction, reasonably match with the experimental observation [Fig. 3(b)]. Note that in Fig. 3(b) we have taken advantage of the symmetry of PDF to map $\theta_{v}$ into the range of 0 to *π*.

The external cues not only determine the average direction to which a cell migrates, but also the uncertainty of its migration direction. To show this, we calculate the differential entropy of $\theta_{v}$ defined as $S_{\theta }=\int_{0}^{2\pi }P_{ss}(\theta )\log_{2}P_{ss}(\theta )d\theta$. As a reference, the maximum entropy $S_{a}$ occurs when $\theta_{v}$ distributes isotropically, such that $S_{a}=\log_{2}2\pi$ bits.

It is particularly informative to examine the uncertainty introduced by varying mechanoguidance. To this end, we set $\frac{\alpha }{D}=1$ and compute $\Delta S_{\theta }=S_{\theta }(\frac{\beta }{D},\theta_{f})-S_{a}$ as shown in Fig. 3(c). For the range of parameters considered here, external cues reduce the uncertainty of $\theta_{v}$, such that $\Delta S_{\theta }<0$. When the strength of mechanochemical cues are fixed (holding $\frac{\alpha }{D}$ and $\frac{\beta }{D}$ constant), entropy of $S_{\theta }$ is minimized when $\theta_{f}=0$, where contact guidance works in concert with chemogradient to reduce the fluctuations of cell migration direction. For fixed chemical guidance, the maximum entropy of $\theta_{v}$ occurs when *α = β* and $\theta_{f}=\frac{\pi }{2}$. In this case, cells are “frustrated” by the perpendicular but equally strong cues, thereby maximizing their directional uncertainty [31].

**FIG. 4.**
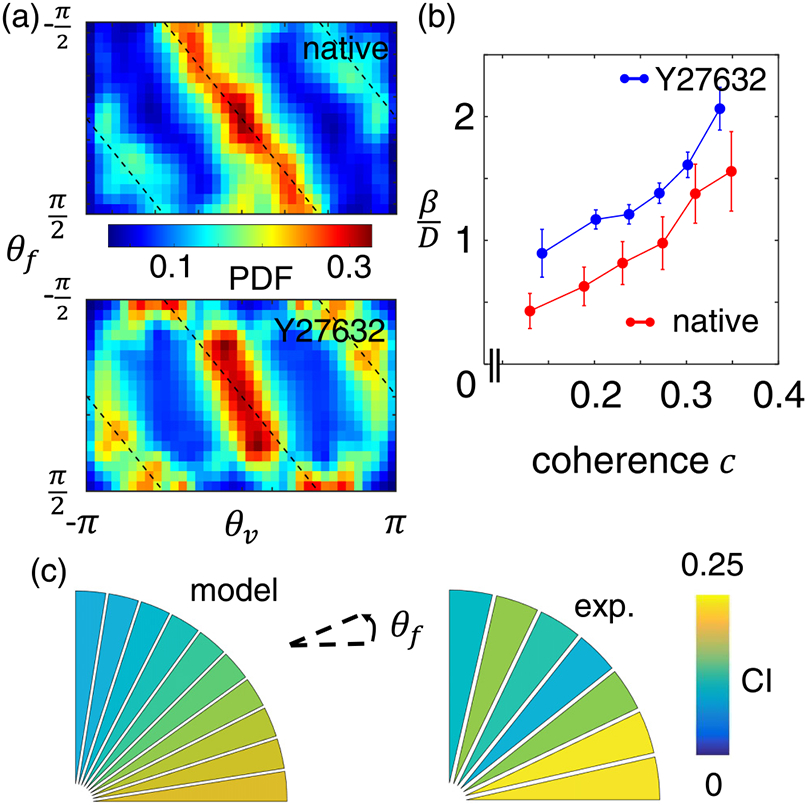
Mechanistic insights into cancer cell motility in response to dual mechanochemical guidance. (a) Experimentally measured conditional probability distribution of $P(\theta_{v}|\theta_{f})$. The data is obtained for cells navigating ECM of relatively high coherence (*c* > 0.2). A Gaussian kernel has been employed to compute the probability distribution. Data in this figure has also been augmented to satisfy the reflection symmetry: for each data point ($\theta_{v},\theta_{f}$), we also include ($-\theta_{v}$, $-\theta_{f}$) in the statistics. Top: native MDA-MB-231 cells. Bottom: cells treated with Rock-inhibitor Y27632. (b) At fixed chemogradient (10% across the device) and ECM approximately in parallel to the $\hat{x}$ axis ($|\theta_{f}|<\frac{\pi }{6}$), model fitting to experimentally measured probability distribution of $\theta_{v}$ reveals the relation between ECM coherence *c* and model parameter $\frac{\beta }{D}$. For native MDA-MB-231 cells the best fit yields $\frac{\alpha }{D}=0.3$ and for Y27632 treated cells the fitting yields $\frac{\alpha }{D}=0.1$. To obtain each fitted data point, a sliding window of *c* with width of 0.1 is used to sample cell velocity at varying ECM coherence. Error bars show the standard deviation of 100 bootstrap results. (c) The chemotactic index ⟨$\cos\;\theta_{v}$⟩ for native MDA-MB-231 cells as the direction of mechanical guidance varies [30]. Here we consider the same condition as in (a). Left: model prediction using fitted parameters as shown in (b). Right: experimental measurements. Abbreviations: CI, chemotactic index; exp., experiment.

Our model predicts *β* to be positively correlated with ECM coherence. Additionally, *β* should also increase if cells become more mechanosensitive to contact guidance cues. To test this prediction, we take advantage of the fact that 3D cell contact guidance is primarily regulated by Rho/Rock signaling [11]. We treat the MDA-MB-231 cells with a well-characterized Rock inhibitor Y27632 and compare the resulting migration directions under 10% serum gradient in collagen matrices. Figure 4(a) shows the conditional probability $P(\theta_{v}|\theta_{f})$ for varying angles of the ECM fibers. For both native and Y27632 cells $P(\theta_{v}|\theta_{f})$ show peaks in the vicinity corresponding to perfect contact guidance [black lines in Fig. 4(a)]. However, the deviations indicate the effect of chemotaxis. In particular, native cells are more responsive to chemical gradient, as the probability near $\theta_{v}=0$ is significantly higher than the probability near $\theta_{v}=\pm \pi$. Y27632-treated cells, on the other hand, have more pronounced peaks near $\theta_{v}=\pm \pi$. These observations suggest that Rho-inhibition suppresses cell response to chemical guidance but enhances response to contact guidance. These observations are also consistent with previous reports on the effects of Rho/Rock inhibition, which promotes the mesenchymal motility phenotype of cancer cells at the expense of the amoeboid mode [32-34] and renders them more sensitive to contact guidance [12].

We next fit experimentally measured conditional probability $P(\theta_{v}|\theta_{f}\approx 0)$ with Eq. (3) to obtain the quantitative relation between mechanochemical cues and model parameters. The fitting results confirm that, while Y27632 treatment reduces cell sensitivity to chemogradient $\frac{\alpha }{D}\approx 0.1$ for Y27632 treated cells compared with $\frac{\alpha }{D}\approx 0.3$ for native cells), Rock-inhibition leads to stronger response to contact guidance [Fig. 4(b)]. These results agree well with our model expectations [29].

Leveraging the quantitative relation between ECM coherence and model parameter, we then elucidate the impact of ECM principal direction to the efficiency of chemotaxis. To this end, we compute for each fiber direction (within equally spaced binning windows) the mean chemotaxis index $CI=\langle \cos\;\theta_{v}\rangle$ under 10% serum gradient and relatively strong coherence (*c* > 0.2). Here the average takes into account experimentally measured distribution of ECM coherence and therefore reflects the effect of ECM structural disorder. The model predicts, and experiments confirm, that as ECM direction rotates from perpendicular to parallel to the chemogradient, the chemotaxis index can increase by as much as twofold [Fig. 4(c)].

## III. DISCUSSION

Tumor metastasis requires cancer cells to navigate through 3D ECM which contains rich mechanochemical cues. In this Letter, we investigate the motility of breast cancer cells which simultaneously experience serum gradient and ECM contact guidance, a combination that mimics key aspects of the physiological microenvironment of tumors [35,36]. Cells following chemotaxis also experience a contact guidance cue that varies spatially in strength and direction, complicating the task of finding an effective path. The cross-talk of external cues, one that is unidirectional and one that is nematic, controls the probability distribution of local cell migration direction. As ECM fibers rotate from perpendicular to parallel with the chemoattractant gradient, the chemotaxis index can increase by more than twofold. We show that, in the presence of dual guidance, impact of ECM alignment is determined externally by the coherence of ECM fibers and internally by cell mechanosensing Rho/Rock pathways. Our results suggest that directed cancer cell migration during metastasis is jointly regulated by biochemical and biophysical cues. Therefore, a comprehensive understanding of tumor microenvironment is imperative to correctly interpret *in vitro* assays and to predict and control cancer cell invasion *in vivo*.

We note that, during the course of metastasis, cancer cells may experience other forms of mechanochemical cues. Growth factors and cytokines, such as TGF-*α* and EGF, are potent chemoattractants. ECM rigidity and porosity also guide cancer cell motility. Interestingly, these mechanochemical cues can be self-generated by cancer cells and their associated stromal cells as they actively remodel the tumor microenvironment [37,38]. We expect the endogenous cues to coordinate complex collective behaviors, which has just begun to be understood [39].

While our phenomenological model takes into account key aspects that determine the fluctuations of cell migration direction, it can be generalized such as by including durotaxis [40] and the driving coefficients may depend on cell morphodynamics [41]. More mechanistic treatments, such as by explicitly considering the physical interaction of cells with fibrous ECM [42,43], could directly predict how the effective parameters are determined by both cell and ECM degrees of freedom.
